# Zn uptake behavior of rice genotypes and its implication on grain Zn biofortification

**DOI:** 10.1038/srep38301

**Published:** 2016-12-02

**Authors:** Sarah E. Johnson-Beebout, Johnvie Bayang Goloran, Francis H. C. Rubianes, Jack D. C. Jacob, Oliver B. Castillo

**Affiliations:** 1 Crop and Environmental Sciences Division, International Rice Research Institute, DAPO Box 7777 Metro Manila, Philippines; 2 Department of Chemical and Biomolecular Engineering, University of Houston, Texas, USA; 3 APOTEX, Incorporated, Toronto, Ontario, Canada

## Abstract

Understanding Zn uptake dynamics is critical to rice grain Zn biofortification. Here we examined soil Zn availability and Zn uptake pathways as affected by genotype (high-grain Zn varieties IR69428 and IR68144), Zn fertilization and water management in two pot experiments. Results showed significant interactions (*P* < 0.05) between genotypes and Zn fertilization on DTPA (diethylenetriaminepentaacetic acid)-extractable soil Zn from early tillering to flowering. DTPA-extractable Zn in soils grown with IR69428 was positively correlated with stem (*r* = 0.78, *P* < 0.01), flagleaf (*r* = 0.60, *P* < 0.01) and g*r*ain (*r* = 0.67, *P* < 0.01) Zn concentrations, suggesting improved soil Zn availability and continued soil Zn uptake by IR69428 even at maturity. Conversely for IR68144, DTPA-extractable Zn was positively correlated only with leaf Zn uptake (*r* = 0.60, *P* < 0.01) at active tillering, indicating dependence on remobilization for grain Zn loading. Furthermore, the highest grain Zn concentration (*P* < 0.05) was produced by a combination of IR69428 and Zn fertilization applied at panicle initiation (38.5 μg g^−1^) compared with other treatments (*P* < 0.05). The results highlight that Zn uptake behavior of a rice genotype determines the fate of Zn from the soil to the grain. This has implications on overcoming Zn translocation barriers between vegetative parts and grains, and achieving grain Zn biofortification targets (30.0 μg g^−1^).

Zinc (Zn) deficiency in human health, which poses a great risk to the cognitive development and physical faculty of many children, is more prevalent in developing countries^1^,^2^. Zn malnutrition is attributed to a lack of access to nutritious food and/or the poor Zn content in staple food, which results in various health problems^3^. Among cereals, rice is characterized as having low grain Zn content and sensitivity to soil Zn deficiency, particularly those under rice paddy cultivation systems^4^. It has been suggested that Zn deficiency is a predominant micronutrient disorder in lowland rice farming systems^5^, thus, biofortification of a major staple food such as rice (*Oryza sativa* L.) is practical and cost-effective in reducing Zn malnutrition among the poor, whose diet mainly depends on rice^6^. Rice grain Zn biofortification is a strategy that complements other strategies (e.g., dietary diversification, supplementation and fortification) aligned with the current global initiative to address micronutrient deficiency issues that lead to human nutritional disorder^7^.

Breeding efforts for rice grain Zn biofortication include conducting trials on root Zn uptake and or grain Zn loading performance of various genotypes^8^,^9^,^10^. Root Zn uptake has been associated with the release of organic exudates from rice roots (see review by Rose *et al*.^11^), which could solubilize or enhance soil Zn availability in the rhizosphere. Although the role of phytosiderophore deoxymugineic acid (DMA) or low molecular weight organic acids in the efficient use of soil Zn in rice plants is unclear^11^, some studies indicate that Zn-efficient genotypes are able to tolerate Zn deficiency and take up Zn as a result of enhanced deoxymugineic acid exudation of phytosiderophore^12^,^13^,^14^. These indicate the potential influence of some genotypes on soil Zn availability and thereby better Zn uptake, particularly in the rice grain. Grain Zn uptake in rice has been suggested to be limited by Zn supply or availability during grain Zn loading period^15^. In Zn-sufficient or surplus conditions, rice plants tend to supply Zn to the grains via root uptake of Zn, whereas in Zn-deficient conditions, grain Zn accumulation is by means of remobilization and root Zn uptake^9^,^16^,^17^. Such uptake pathway of Zn in rice is also shown in the model proposed by Sperotto^17^, which highlights the differences in grain Zn accumulation based on Zn supply conditions. Most of the mentioned studies, however, were conducted in nutrient solution (Jiang *et al*.^16^, Wu *et al*.^9^, Impa *et al*.^9^), which may not reflect the known uptake behavior of high grain Zn genotypes when grown in soils (either in pot or field experiments) due to differing soil environments that can affect both soil Zn status and plant growth^9^. The present study noted results from Wissuwa *et al*.^4^, who concluded that grain Zn concentration is largely determined by genotype rather than by Zn fertilization, which could be attributed to differences in Zn uptake behavior. In addition, Tuyogon *et al*.^10^, who used both IR68144 and IR69428 in field experiments, observed a significant increase in DTPA-extractable soil Zn and grain Zn uptake in longer-duration genotypes as a result of water management (WM), such as by alternate wetting and drying (AWD). These authors concluded, however, that the effect of AWD on grain Zn concentration was still minimal and the effect of Zn-fertilization (ZF) timing on grain Zn accumulation was not significant. These suggest that drying events and ZF should be optimized to correspond with the crop demand for Zn, particularly during grain Zn loading, which may vary among rice genotypes.

Agronomic practices, such as WM and ZF, are established strategies for improving rice yield^18^, which are also considered complementary strategies for Zn biofortication in rice^19^,^20^,^21^. Both WM and ZF are employed to improve soil Zn availability that could meet plant demand for Zn^22^,^23^. Water management strategies, such as AWD, expose soils to both aerobic and anaerobic conditions that change soil pH, resulting in either more or less available soil Zn to plants. Under anaerobic conditions, soil pH tends to increase or decrease to near neutral driven by changes in carbonate equilibria^24^. In addition, ZF may become irrelevant due to the immobilization of applied Zn fertilizer, resulting in the unavailability of Zn to plants. A recent report revealed that rapid and irreversible immobilization of applied Zn fertilizer occurs after submergence^25^. This suggests that ZF should be combined with drainage to ensure aerobic conditions when the fertilizer is applied to soils. This further implies that, if drainage and ZF are employed simultaneously in times when plants greatly demand Zn for physiological or reproductive development, grain Zn accumulation can be expected. However, this may still depend on genotypic response to agronomic management. To date, reports remain limited in relation to genotype variations in Zn uptake pathways.

This study hypothesizes therefore that WM (drying) and ZF should be employed simultaneously to increase available Zn in the soil: (H1) in the mid-season (i.e., active tillering or panicle initiation) when the plants’ demand for Zn may be greater in preparation for the reproductive stage, and (H2) in the late-season so that plants could have enough supply of Zn to meet the grain Zn loading requirement. This was carried out in two pot experiments. The first used a single high grain Zn genotype, IR69428, to examine the effect of the timing of ZF on grain Zn content and Zn uptake performance relative to plant growth stage and flooding/draining periods. To broaden the assessment of these hypotheses, a second pot experiment was conducted using IR69428 plus an additional genotype, IR68144. The timing of WM and ZF in experiment 1 was modified as specified in these additional hypotheses: (H3) D1 (1 week drying before ZF at active tillering) allows for similar plant uptake or an increase in available soil Zn as much as D2 (1 week drying following ZF at active tillering); (H4) both D1 and D2 are better than D0 (no drying, continuously flooded) because the redox potential stays high for some time after reflooding and (H5) ZF at panicle initiation with D3 (1 week drying before ZF at panicle initiation) and D4 (1 week drying after ZF at panicle initiation) would be better than D1 and D2 because the rhizosphere would then be more oxidized. Here, we used two high grain Zn genotypes to examine the contrasting (1) Zn uptake behavior, (2) relationship between soil Zn and plant Zn uptake dynamics and (3) pathways, including their effect on soil Zn availability, and to examine the combined effect of ZF and WM options in determining optimum drying relative to early ZF and optimum timing of late ZF to simultaneously achieve agronomic Zn sufficiency and high grain Zn.

## Results

### Experiment 1: Effects of water management and Zn fertilization

#### Soil Zn availability

**Figure 1:**
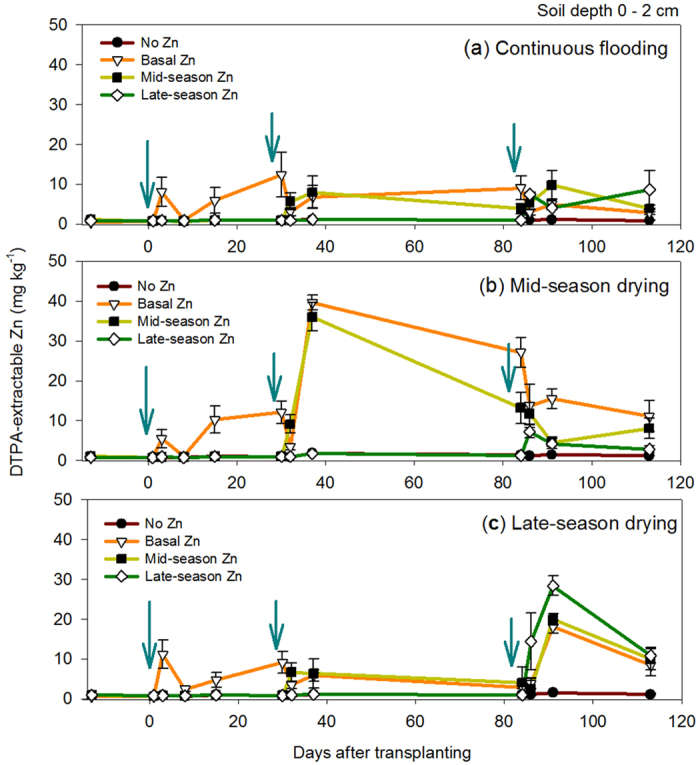
Concentration of soil Zn at 0 to 2 cm soil depth throughout plant development in Exp. 1, showing each water management treatment: (**a**) continuous flooding, (**b**) mid-season drying, and (**c**) late-season drying. Arrows indicate the time of Zn application for (**a**) basal Zn: a day before transplanting, (**b**) mid-season Zn: 30 DAT and (**c**) late-season Zn: 85 DAT. Values are the average ± standard error (*n* = 4). DTPA-extractable Zn levels in soils prior to the start of the study is below the critical levels (0.8 mg kg^−1^) suggested by Dobermann and Fairhurst^26^.

**Figure 2:**
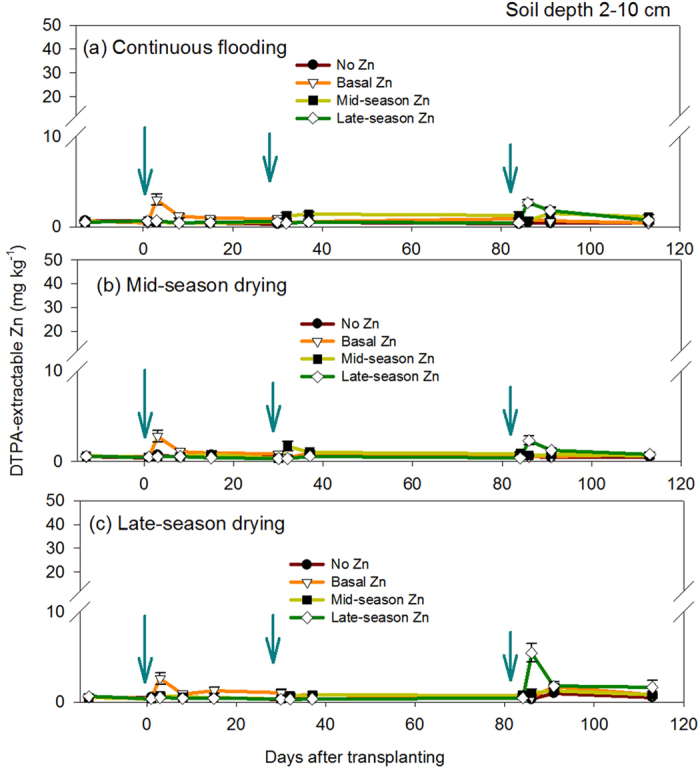
Concentration of soil Zn at 2 to 10 cm soil depth throughout plant development in Exp. 1, showing each water management treatment: (**a**) continuous flooding, (**b**) mid-season drying, and (**c**) late-season drying. Arrows indicate the time of Zn application for (**a**) basal Zn: a day before transplanting, (**b**) mid-season Zn: 30 DAT and (**c**) late-season Zn: 85 DAT. Values are the average ± standard error (*n* = 4). DTPA-extractable Zn levels in soils prior to the start of the study is below the critical levels (0.8 mg kg^−1^) suggested by Dobermann and Fairhurst^26^.

The DTPA-extractable Zn concentrations at soil depths: 0–2 m (0.67 ± 0.08 mg kg^−1^) and 2–10 cm (0.53 ± 0.09 mg kg^−1^) prior to the start of the experiment were below the critical Zn level (0.8 mg kg^−1^) suggested by Dobermann and Fairhurst^26^. The availability of DTPA-extractable Zn throughout the growing period of rice was higher at 0–2 cm than 2–10 cm soil depth (Figs 1 and 2). Results showed greater availability of DTPA-extractable Zn under mid-season drying (MSD) compared with other water management treatments (Fig. 1). Zn-fertilization treatments such as basal Zn and mid-season Zn under MSD, ranging from 3.01–39.5 mg kg^−1^ and 8.09–35.2 mg kg^−1^, respectively (Fig. 1), both showed greater availability of DTPA-extractable Zn than that of no Zn. At a soil depth of 0–2 cm, there were significant effects of individual factors (ZF and WM) observed on DTPA-extractable Zn during active tillering (32–37 DAT); significant interaction effects of ZF and WM were observed at the later stages of growth (i.e., 87–91 and 113 DAT) (Table S1). At a soil depth of 2–10 cm, DTPA-extractable Zn was low, ranging from 0.25–5.54 mg kg^−1^ (Fig. 2). There was an increase in DTPA-extractable Zn in a specific period of time that corresponded to the time of Zn fertilizer application (Fig. 2). The effect of ZF on DTPA-extractable Zn at a soil depth of 2–10 cm was significant on all stages of growth, whereas the effect of WM was only significant during grain-filling (87–91 DAT) (Table S1).

#### Plant Zn, Fe and P concentration

**Figure 3:**
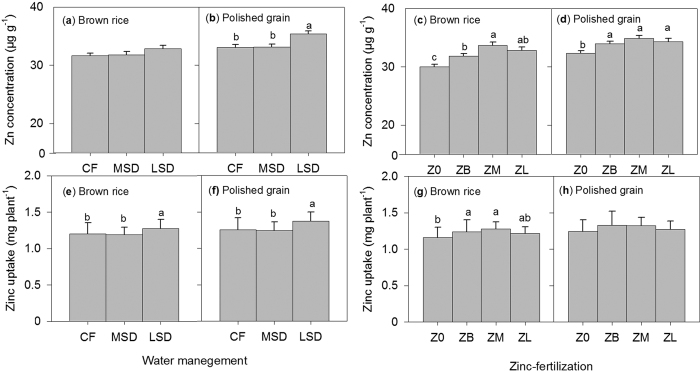
Grain Zn concentration (**a–d**) and uptake (**e–h**) of brown rice and polished grain in Exp. 1, showing the effects of Zn fertilization: no Zn (Z0), basal Zn (ZB), mid-season Zn (ZM), and late-season Zn (ZL) and water management continuous flooding (CF), mid-season drying (MSD) and late-season drying (LSD). Values are the average ± standard error: ZF (*n* = 12) and WM (*n* = 16). ANOVA is presented in Table S2. Different letters indicate significant differences among treatments (LSD, *P* < 0.05).

Regardless of treatment (genotypes, Zn fertilization and water management), Zn concentrations and uptake in different parts of the rice plant were observed to be significantly (*P* < 0.01) higher in the stem (Table S3 and S4) than in other plants parts. The effect of ZF on stem Zn concentration was significant from the early stage of growth up to the harvest, whereas for WM, stem Zn concentration was only statistically significant during the grain-filling stage (86–91 DAT) (Table S2 and S3). ZF and WM only showed significant interaction on stem Zn concentration measured at late maturity (113 DAT) (Table S2). For grain Zn concentration and uptake, significant interaction between ZF and WM was not observed but it did show significant individual factor effects. Results revealed that late-season drying (LSD) had higher grain Zn (polished grain) concentration and uptake than continuous flooding (CF) and MSD under WM treatments (Fig. 3e,f), whereas under ZF treatments, significant differences in grain Zn concentration (brown rice and polished grain) were observed only between Zn-fertilized [basal Zn (ZB), mid-season Zn (ZM) and late-season Zn (ZL)] and unfertilized (no added Zn) treatments (Fig. 3c,d). Results further revealed significant correlation coefficients between brown rice Zn concentration and DTPA-extractable soil Zn measured during grain-filling (86–91 DAT), either at 0–2 cm (*r* = 0.70, *P* < 0.01) or 2–10 cm (*r* = 0.54, *P* < 0.05) soil depth under LSD (Table 1).

**Figure 4:**
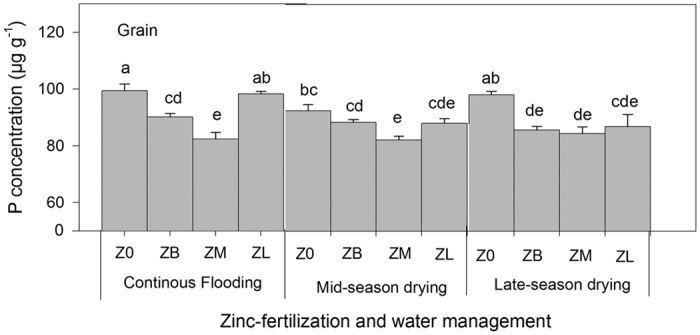
Phosphorus (P) concentrations (Exp. 1) in rice grain showing the significant interaction between water management and Zn fertilization. Values are the average ± standard error: WM (*n* = 16) and WM x ZF (*n* = 4). ANOVA can be found in Table S5.

The concentration of other nutrients such as Fe and P in parts of the rice plant was also measured in the study. Fe concentration was not significantly affected by WM and ZF at late maturity, whereas P concentration showed a significant influence of both WM and ZF treatments, particularly in the grain, stem and leaf (Table S5). Interestingly, for grain P concentrations, results showed significant interactions by both WM and ZF (Table S5), in which a combination of CF and no Zn fertilizer had the highest grain P (Fig. 4).

### Greenhouse Experiment 2: Effects of water management, Zn fertilization and genotypes

#### Soil Zn availability

**Figure 5:**
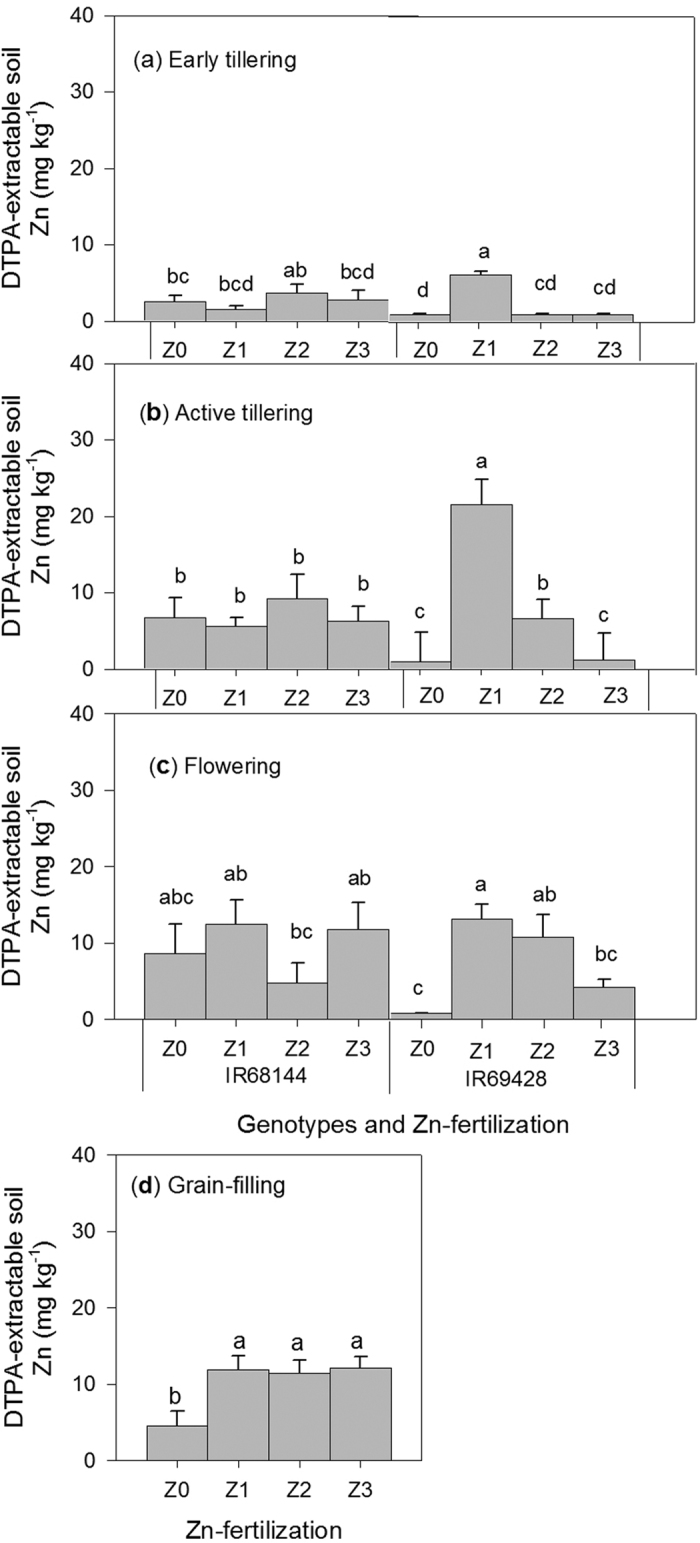
Interaction effects of genotypes and Zn fertilization (Exp. 2) on DTPA-extractable soil Zn measured at different times: (**a**) early tillering (7-25 DAT), (**b**) active tillering (26-40 DAT), (**c**) flowering (69-83 DAT) and (**d**) effects of Zn fertilization on soil Zn during grain-filling. No Zn (Z0), Zn at active tillering (Z1), Zn at panicle initiation (Z2), Zn at early heading (Z3). Values are the average ± standard error: genotypes x Zn fertilization (*n* = 5) and Zn fertilization (*n* = 10). ANOVA is presented in Table S6.

Our results showed significant (*P* < 0.05) effects on interaction between genotype and Zn fertilization on the concentrations of DTPA-extractable soil Zn throughout the growth season (7–25, 26–47 and 69–75DAT), except for the late-maturity stage (Fig. 5, Table S6). The DTPA-extractable Zn under treatment combination Z1 (Zn applied at active tillering) and IR69428, measured at 26–40 DAT, showed the highest concentration compared with other treatment combinations (Fig. 5b). The results further showed that DTPA-extractable soil Zn remained higher at the grain-filling stage in Zn-fertilized treatments (Fig. 5).

#### Zn uptake and concentration in the rice grain and other parts

**Figure 6:**
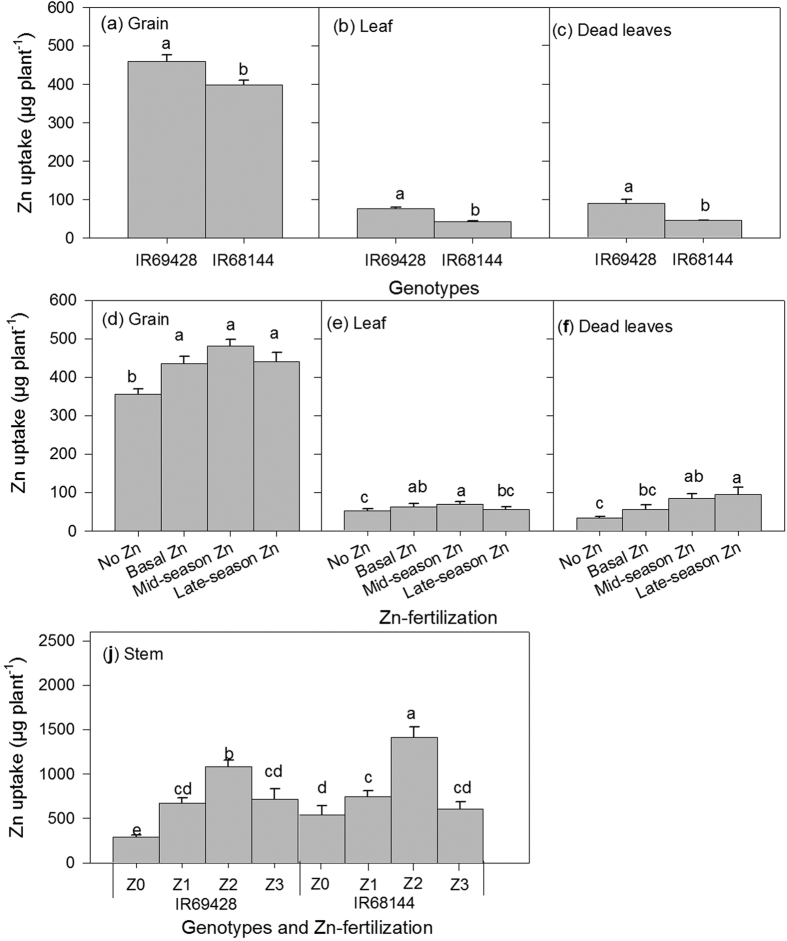
Effects of genotypes and Zn fertilization (Exp. 2) on Zn uptake in various parts of rice plants at maturity, showing only significant main effects and the interactions (P < 0.05). Error bars represent standard error: genotypes (*n* = 20), Zn fertilization (*n* = 5) and genotypes x Zn fertilization (*n* = 5). ANOVA is presented in Table S7.

**Figure 7:**
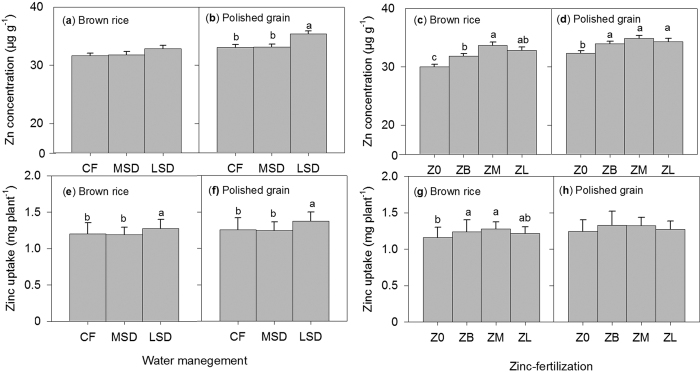
Interaction effects of genotypes and Zn fertilization (Exp. 2) and or water management on grain Zn concentration. Values are the average ± standard error: genotypes x Zn fertilization (*n* = 5) genotypes x WM (*n* = 4). ANOVA is presented in Table S7.

The ANOVA for effects of treatments such as ZF (*P* = 0.0875), WM (*P* = 0.1274) and genotypes (*P* = 0.1587) on grain yield [IR69144 (12.7 ± 0.35 g hill^−1^) and IR69428 (12.3 ± 0.29 g hill^−1^) were not significant. The effects of ZF, WM and genotype however, on Zn uptake varied significantly among rice plant parts (Table S7). All the individual effects of ZF, WM and genotype were observed significantly on the uptake of Zn by the stem, leaves and panicle during early heading, but there were no effects on interaction (Table S7). Results also showed that the highest Zn uptake was in the stem followed by the panicle and leaves (Table 2). At late maturity, the interaction effect of the treatments (ZF x WM and ZF x genotype) on plant Zn uptake were only significant on the stem, whereas, individual effects (*P* < 0.05) of treatments such as genotype and ZF (Table S7) were shown on the other parts, particularly grain Zn uptake. Specifically, IR69428 was found to significantly (*P* < 0.05) take up more Zn in the grain and leaves compared with IR68144 (Fig. 6a–c). The effect of ZF was only significant for grain Zn between treatments with and without Zn fertilizer (Fig. 6d), but significant differences were shown in the effects of ZF on the leaves (Fig. 6e,f). Also, stem Zn uptake was the highest under mid-season Zn and IR68144 treatment (Fig. 6j). On the other hand, the effects of treatment interaction on grain Zn concentration appeared to be more affected (*P* < 0.01) by genotype interaction (i.e., ZF x genotype and WM x genotype) rather than by ZF or WM interactions with other treatments (Table S7). Interactions between IR69248 x Z2 (Zn applied at panicle initiation) or Z3 (Zn applied at early heading) and IR69428 x D2 resulted in the highest grain Zn concentrations compared with other treatment combinations (Fig. 7). In addition, the concentrations of Zn in the grain (*r* = 0.67, *P* < 0.01), stem (*r* = 0.78, *P* < 0.001), leaf (*r* = 0.46 *P* < 0.05), dead leaves (*r* = 0.65, *P* < 0.01) and flagleaf (*r* = 0.60, *P* < 0.01) of IR69428 showed significant correlations with DTPA-extractable soil Zn (Table 3), whereas IR68144 did not show such relationships during the grain-filling stage. Furthermore, in maturity, relationships between the Zn concentration or Zn uptake in grain Zn and other plant parts (stem, leaf, flagleaf and dead leaves) were all significant except for grain and flagleaf Zn concentration (Table 4).

## Discussion

Our results demonstrate that MSD and mid-season ZF significantly increased DTPA-extractable Zn at 0–2 cm soil depth, which was higher than that of CF and LSD treatments despite the addition of the same amount of Zn fertilizer. The concentrations of DTPA-extractable Zn under MSD in our pot experiments (0–2 cm soil depth) were up to 8 times higher than the values reported by Tuyogon *et al*.^10^ for field experiments at 0–2 cm soil depth under AWD and ZF at 10 kg Zn ha^−1^. This may be attributed to the combined effect of MSD and mid-season ZF on soil Zn availability at a shallow depth (0–2 cm), which confirms our hypothesis (H1). At lower soil depths (2–10 cm), the concentrations of DTPA-extractable Zn was significantly the highest under LSD and late-season Zn (Fig. 2c), which partially confirms our hypothesis (H2). These results indicate that, during the vegetative stage, soil at a 0–2-cm depth is critical to soil Zn availability in plants, and the 2–10-cm depth becomes more important as a source of Zn during the stages of flowering or grain filling, which might be due to effects of drying, ZF, and an established root system, which is more efficient in Zn uptake in deeper soils (Brahim *et al*.^27^). On the other hand, the effects of WM treatments on increasing DTPA-extractable Zn were translated into significant correlations of stem and leaf Zn concentration with DTPA-extractable Zn either in 0–2-cm or 2–10-cm soil depth during MSD: 32–37 DAT (Table 1), suggesting predictability of soil Zn uptake by rice plants during the drying period. However, a significant relationship was also found in LSD and CF treatments at the 2–10-cm soil depth (Table 1). This suggests that, irrespective of WM treatments, the rice plants (IR69428) in this study were able to take up soil Zn effectively even in oxidized (MSD and LSD) or reduced (CF) soil conditions.

The ability of genotype IR69428 to take up soil Zn effectively is supported by the results from experiment 2 for DTPA-extractable soil Zn measured during the vegetative growth stage (26–47 DAT) (Fig. 5). Results showed that DTPA-extractable Zn concentrations in soils grown with IR69428 (22 mg kg^−1^) were significantly higher than that with IR68144 (6.0 mg kg^−1^) despite receiving the same ZF (i.e., mid-season Zn) treatment (Fig. 5b). Zn uptake by plants has been associated with several root-related processes such as the release of low molecular weight organic acids and the efflux of phytosiderophores to solubilize unavailable forms of Zn in soils^9^,^13^. This may be used by IR69428 to mobilize soil Zn, which deserves a future investigation. In experiment 2, no significant effects were observed for WM treatments on DTPA-extractable Zn, rather, it was the interaction effect of genotypes and ZF that was consistently significant from active tillering to flowering (Table S6). It was also noted that at these growth stages (tillering and flowering) the concentrations of DTPA-extractable Zn in the treatments without Zn fertilizer were above the critical levels (e.g. IR68144) suggesting genotypic influence (Fig. 5a,b,c). A stronger influence of genotypes rather than WM was also demonstrated by strong correlations between DTPA-extractable soil Zn and plant (stem and leaf) Zn uptake for IR69428 than for IR68144. This was measured at 26–47 DAT, 69–75 DAT and 83–100 DAT (Table 3). In brief, the relationships between DTPA-extractable Zn and plant Zn uptake in various plant organs of IR69428 were consistent with the results in experiment 1 using the same genotype (Table 1) despite the differences in WM and ZF timing. Our results demonstrate that, in addition to the timing of drying and ZF, genotypes played a significant role in altering soil Zn availability, resulting in better Zn uptake and utilization as a result of the continued Zn uptake behavior beginning from active tillering to maturity shown by IR69428 (Tables 1 and 3). Further investigation is necessary to understand specific mechanisms used by high grain Zn genotypes in mobilizing soil Zn and plant Zn uptake in contrasting soils, as rhizosphere-related processes vary depending on several environmental factors such as soil types, nutrient availability and genotype^9^,^28^.

Experiment 1 results demonstrate significant individual effects of WM and ZF not on brown rice Zn concentration but on polished grain Zn concentration. The effects of WM (i.e., during LSD) on polished grain concentration were stronger than ZF. This is because under ZF, the significant differences can only be observed between plants with Zn and without Zn fertilizer (Fig. 3), but not between plants with different ZF timing. Specifically, results showed that LSD had a significantly higher grain Zn concentration (35.40 μg g^−1^) compared with CF (33.01 μg g^−1^) and MSD (33.15 μg g^−1^) (Fig. 3), suggesting the beneficial effect of LSD on grain Zn loading for genotype IR69428. Our results slightly differ from those of Tuyogon *et al*.^10^, who reported that AWD had a positive effect on grain Zn concentration but that the contributed increase was minimal (2.0 μg g^−1^); hence, the desired grain Zn concentration (30.0 μg g^−1^) was not achieved. This discrepancy could possibly be due to variation in plant uptake responses to controlled environment conditions that do not often represent prevailing field conditions (Trijatmiko *et al*.^29^). Furthermore, our results are supported by positive relationships between grain Zn concentrations (either in brown rice or polished grain) and DTPA-extractable soil Zn (0–2 cm soil depth) under LSD (Table 1), demonstrating not only continued root uptake of soil Zn even at late maturity but also Zn translocation to the grains for IR69428. Our results in experiment 2 further provide a clear picture of this behavior or mechanism employed by IR69428 for Zn uptake. Meanwhile, our hypotheses relating to the effects of WM on DTPA-extractable soil Zn at different growth stages, particularly for H3 (at active tillering), H4 (active tilering) and H5 (panicle initiation), did not show significant effects (Table S6). This was also reflected in the effect of WM on grain Zn uptake at maturity (Table S7). However, our results suggest that the H3, H4 and H5 hypotheses are partially confirmed in the interaction effects between WM and genotypes (Table S7), which have significantly contributed to grain Zn biofortication in this study.

Correlation coefficients between grain Zn concentration/uptake and soil Zn revealed contrasting results for the two genotypes even though both were high-grain Zn lines (Table 3). Stem, leaf and grain Zn concentration and uptake by IR49428 consistently showed positive significant relationships with DTPA-extractable Zn from active tillering to maturity, whereas IR68144 showed such a relationship only in leaf Zn uptake during the early stages of growth (26–47 DAT) (Table 3). This indicates that the former genotype relies strongly on continued and direct Zn uptake via its roots for grain Zn loading, whereas the latter genotype relies more on translocation from other parts into the grain. In a nutrient solution study involving IR69428, this genotype demonstrated continued root Zn uptake until maturity, particularly in Zn-sufficient conditions, but there was no Zn translocation observed from the roots to the grains^9^. Our results for soil-based experiments 1 and 2 indicated a similar behavior for IR69428 (continued Zn uptake during grain-filling) to that of Impa *et al*.^9^, with significantly positive correlations between DTPA-extractable soil Zn and Zn in plant parts from active tillering to late maturity (Table 3). But, unlike in Impa *et al*.^9^, we also observed Zn translocation in grains as shown in significant correlations between concentrations of grain Zn and Zn in plant parts (Table 4).

The observed relationships between DTPA-extractable soil Zn and Zn in plant parts (Tables 1 and 3) also imply Zn translocation in various plant parts (e.g., stem, leaf and grains) that occurred regardless of WM and ZF treatments. While significant relationships between DTPA-extractable soil Zn and stem or grain Zn were not observed for IR68144, Zn uptake and concentrations between plant parts (flagleaf, leaf, dead leaves and stem) and grain were all positively correlated, suggesting remobilization of Zn from other parts into the grain, which is in agreement to the report for genotype IR68144^8^. It has been reported that Zn remobilized from plant parts to the grain is replaced by Zn taken up by the roots in order to maintain plant organ requirements for Zn^30^. This shows that Zn remobilized from other organs to the grain can be compensated by root uptake, which could explain the positive correlations observed between plant part Zn and grain Zn, either in uptake or concentrations (Table 4). Interestingly, IR69428 also showed positive correlations between plant part Zn and grain Zn, with the highest correlation coefficient (*r* = 0.82–0.88, *P* < 0.01) observed between stem Zn and grain Zn (Table 4). This suggests that IR69428 may be using both continued Zn uptake and Zn translocation mechanisms for grain Zn loading. This could be possible because the minimum requirement of plant parts for Zn can be compensated by the available supply of Zn in soils [i.e., late-season ZF and LSD both improves Zn availability to plants (Fig. 1)] and continued Zn uptake by IR69428 even at maturity. Given this condition, IR69428 would be more likely to benefit from LSD and late-season ZF than IR68144, which implies that the contribution of WM and ZF to rice grain Zn biofortification may be determined by the Zn uptake behavior of rice genotypes. These findings warrant further investigation under field conditions with contrasting soils or seasons.

The rice stem is regarded as a critical part in nutrient transport between vegetative and reproductive tissues^30^. The present study also observed that substantial amounts of Zn in both genotypes were stored in the stem. Stem Zn uptake for IR68144 was significantly higher than in IR69428 (Table 2). This, however, did not mean higher grain Zn concentration. For example, IR69428 tended to translocate more Zn from the stem to the grain because Zn that moved out of the stem could be replaced or compensated by the available supply of soil Zn (e.g., LSD and late-season Zn) and continued uptake of Zn through the roots. Hence, there was lower stem Zn concentration in IR69428 though it had higher grain Zn than IR68144. Conversely, IR68144 appeared to regulate the translocation of Zn from the stem to the grain because it was maintaining the minimum organ Zn requirement as indicated in the works of Stomph *et al*.^30^. The case of IR68144 may represent the bottleneck in grain Zn biofortification highlighted in previous studies (e.g., Stomph *et al*.^30^, Colangelo and Guerinot^31^, Sperotto *et al*.^32^), which could be overcome if high grain Zn genotypes employed both these traits: a) continued Zn uptake by roots throughout grain-filling and b) Zn translocation from leaves to grain as shown by IR69428 in this study.

This study also observed significant changes in phosphorus (P) and iron (Fe) concentrations in different plant parts in both genotypes, which were mainly driven by WM. Grain P concentration under CF and no ZF was the highest among treatments, which demonstrated the known inverse relationship between P and Zn uptake (Mandal *et al*.^33^, Das *et al*.^34^). Pathways of Zn from soil to the grain for these high-grain Zn genotypes should therefore be investigated in contrasting soils with emphasis on soil P and Zn interactions. Meanwhile, our results for Fe uptake by plants showed two key points: first, CF is known to increase Fe in the soil solution through reductive dissolution of Fe oxides, which explains Fig. 4a,b. Second, plants must be otherwise regulating Fe that reaches the grain because no difference in grain Fe concentration was observed (Table S5), which is promising because it indicates that WM that benefits Zn biofortification did not hurt Fe biofortification.

## Conclusions

Overall, our results demonstrated that DTPA-extractable soil Zn concentrations were altered by genotype in addition to WM and ZF. The continued root uptake of soil Zn by IR69428, even during grain-filling was exhibited by significant positive correlations between DTPA-extractable soil Zn and Zn concentration in multiple plant parts (e.g., stem, flagleaf, leaf and grain). This continued root uptake of Zn resulted in greater grain Zn concentration compared with IR68144, which tends to rely on Zn translocation as a mechanism for grain Zn loading. The continued uptake of soil Zn by IR69428 complements well with LSD and late-season ZF due to benefits of having a greater supply of soil Zn during grain Zn loading. This study also highlights that, in addition to WM and ZF, Zn uptake behavior of rice genotypes determines the fate of Zn from the soil to the grain, which have implications for overcoming the bottleneck in rice grain biofortification. These results however should be verified under field conditions at contrasting soil environments and seasons to further determine Zn uptake behavior of high grain Zn genotypes.

## Materials and Methods

### Soil preparation, seed sources and plant management

Pot experiments were carried out in a greenhouse at the International Rice Research Institute, Los Baños, Laguna, Philippines. The top 20 cm of the soil was removed from field UG3 (latitude N 14° 08′ 78.5″ and longitude E 121° 16′14.2″ longitude) at the IRRI upland farm and taken to the greenhouse. Pots (bottom length: 43 cm, bottom width: 30 cm, top length: 50 cm, and top width: 35 cm) were filled with 10.5 kg of soil (fresh weight) and moisture content (MC) was measured. These were then flooded in order to maintain a reduced condition during homogenization prior to the start of the experiment. Rice straw was cut into 5-cm pieces and was added to the soils 2 weeks prior to transplanting. The amount of rice straw added (3.1 g kg^−1^ pot^−1^) was based on soil dry weight (i.e., samples were oven-dried at 105 °C up to constant weight) in both experiments. In experiment 1, NPK fertilizer (180 g N + 40 g P + 40 g K kg^−1^) was obtained from the IRRI Experiment Station. N was applied at 3 applications (basal, active tillering and panicle initiation), whereas P and K were applied at basal stage only. In experiment 2, basal fertilization with N (0.36 g pot^−1^), P (0.51 g pot^−1^), and K (0.69 g pot^−1^) was done a day prior to transplanting into the pots. To ensure better vegetative growth, 0.36 g pot^−1^ of N fertilizer was added during active tillering and panicle initiation.

The seed used for experiment 1 was IR69428-6-1-1-3-3 (*Tropical japonica*), a high-grain Zn genotype^10^ with a relatively long duration (94 days to flowering). In experiment 2, both IR69428-6-1-1-3-3 and IR68144-2B-2-2-3-1-127 (released as variety MS13 in the Philippines in 2003) (*Indica*) were used to represent a contrast between indica and japonica with different genetic heritages. The abbreviated names used in the rest of the manuscript are IR69428 and IR68144. Both are promising lines for high Zn accumulation in grains^10^ and were obtained from the Plant Breeding, Genetics and Biotechnology Division of IRRI. Unlike IR69428, IR68144 had a shorter duration (81 days to flowering). Thirteen-day-old seedlings were transplanted into pots with 2 seedlings per hill (details in experiments 1 and 2). Pest and disease control included hand removal of snails from the pots and mosquito netting placed over seedlings in the nursery and pots for 3 weeks following transplanting to control green leafhopper, thereby preventing tungro.

### Experiment 1

This experiment examined the effect of the timing of Zn fertilizer application on grain Zn content, relative to plant growth stage and flooding or draining periods. The different levels of Zn fertilizer included in the experiment are: no Zn (Z0), basal Zn (ZB: applied by top dressing into the floodwater one day after transplanting), mid-season Zn (ZM: applied at the beginning of the mid-season drying period), and late-season Zn (ZL applied at the beginning of the late-season drying period). All Zn fertilizer was added at 20 kg ha^−1^ Zn in the form of reagent-grade zinc sulfate heptahydrate dissolved in 200 mL deionized water and applied into at least 1 cm standing floodwater, spread evenly over the surface of the water in the pot.

Three levels of WM were included in the experiment, namely, continuous flooding (CF), mid-season drying (MSD), and late-season drying (LSD). Under CF, the soils were flooded at a 5-cm depth for the duration of the season. In MSD, the soils were allowed to dry for one week during the late tillering stage and kept flooded at other times; in LSD, the soils were allowed to dry for one week, starting at one week after flowering, and then re-flooded to 5 cm just before harvest. Drying in MSD and LSD treatments referred to in this study means that soils were kept below −10 kPa soil matric potential. Irrigation was done with reverse osmosis water to avoid problems between Zn and Fe. The 12 treatment combinations were arranged in randomized complete block design with 4 replications.

### Experiment 2

This experiment was performed using a three-factor [4 (ZF) x 5 (WM) x 2 genotypes = 40 treatment combinations] laid out in randomized complete block design with 3 replicates. First, ZF included No Zn (Z0), Zn applied at active tillering (Z1), Zn applied at panicle initiation (Z2), and Zn applied at early heading (Z3). The same protocol for Zn fertilization in experiment 1 was used here. Second, WM included continuous flooding or no drying (D0: pots were flooded continuously throughout the experiment to a depth of 5 cm standing floodwater), 1 week drying before Zn fertilization at active tillering (D1: pots were allowed to dry via evapotranspiration a week prior to Zn application at active tillering, and then re-flooded to 5 cm at the time of Zn fertilization), 1 week drying after Zn fertilization at active tillering [D2: pots were allowed to dry for one week following Zn fertilization at active tillering (similar to the first experiment), and then re-flooded to 5 cm], 1 week drying before Zn fertilization at panicle initiation (D3: pots were allowed to dry for one week prior to Zn fertilization at panicle initiation, and then reflooded to 5 cm at the same time as Zn fertilization), 1 week drying after Zn fertilization at panicle initiation (D4: pots were allowed to dry for one week following Zn fertilization at panicle initiation, and then reflooded to 5 cm). Third, genotypes had two high-grain Zn rice genotypes, including IR69428 and IR68144.

### Soil sampling and analyses

The physicochemical properties measured were pH (6.0 ± 0.02) as described by Thomas^35^, texture (clay: 38.83% ± 0.16; sand: 16.5% ± 0.22; silt: 44.66% ± 0.21) by a hydrometer^36^, organic C (1.4% ± 0.01) by potassium dichromate^37^, Olsen phosphorus (21.5 mg kg^−1^ ± 0.22) by sodium bicarbonate^38^ and cation exchange capacity (27.5 cmol_*c*_ kg^−1^ ± 0.23) by ammonium acetate pH 7^39^, including exchangeable K (1.04 cmol_*c*_ kg^−1^ ± 0.01), Mg (8.93 cmol_*c*_ kg^−1^ ± 0.06), and Ca (15.4 cmol_*c*_ kg^−1^ ± 0.16).

Soil sampling and measurements of plant-available soil Zn were done at different times: 1–30 (prior to MSD), 30–37 (MSD period), 86–97 and 113 days after transplanting (DAT) for experiment 1; and 7–25 (D1 = 1 week drainage before Zn application at active tillering), 26–47 (D2 = 1 week drainage after Zn application at active tillering), 60–75 (D3 & D4 = 1 week drainage before and after Zn application at panicle initiation, respectively) and 83–110 DAT for experiment 2. In experiment 1, plant-available Zn was measured at two depths: 0–2 cm and 2–10 cm, whereas for experiment 2, the Zn measurement was done only at 0–2 cm. Plant-available soil Zn was measured by wet DTPA suitable for flooded soils as described by Johnson-Beebout *et al*.^40^. The DTPA extracts were analyzed for Zn by atomic absorption spectroscopy (AAS).

### Plant sampling and analyses

Destructive plant sampling was carried out in both experiments. This was done at times that corresponded to soil sampling. For experiment 1, plant sampling was conducted on: (1) transplanted seedlings, just before basal Zn fertilization, (2) one week after basal Zn fertilization (same day as soil sampling), (3) two weeks after transplanting, (4) just before MSD and Zn-fertilization, (5) at the end of MSD (1 week after Zn fertilization, before reflooding), (6) just before LSD and Zn fertilization, (7) at the end of LSD (1 week after Zn fertilization, before reflooding) and (8) at seed harvest time. For experiment 2, destructive plant sampling was conducted one week after Zn fertilization: (1) at active tillering, (2) panicle initiation, (3) early heading and (4) harvest time.

Total above-ground biomass (dry weight) of leaves, stems, flag leaves, panicle and grain was measured, and each plant part was subjected to acid digestion using a nitric perchloric mixture (HNO_3_: 0.16 mol L^−1^ and HClO_4_: 0.28 mol L^−1^) as described by Tuyogon *et al*.^10^. Acid digests were determined for total Zn by inductively coupled plasma-optical emission spectroscopy analysis (PerkinElmer Inc.-Optima5300DV, Waltham, MA).

### Statistical analyses

For DTPA-extractable soil Zn data, which were measured at different growth stages from early stage to maturity, we grouped the data sets together based on the following days: 1–30 (prior to MSD), 32–37 (MSD period), 86–91 and 113 DAT (experiment 1) and 7–25, 26–47, 60–75 and 83–110 DAT (experiment 2). We then calculated the means of each group for DTPA-extractable soil Zn to represent the available soil Zn for that particular period or stage of rice growth.

A Pearson correlation analysis was performed between the mean DTPA-extractable soil Zn and Zn concentration in the stem, leaf, flagleaf, panicle and grain. Soil and plant Zn data sets were checked for normality prior to Analysis of Variance (ANOVA). For experiment 1, a two-way (by ZF and WM) ANOVA was used, and for experiment 2, a three-way (by ZF and WM and genotypes) ANOVA was used. The treatment mean differences were calculated at 5% level of significance using a least significant difference test. All statistical analyses were performed using STATISTIX 8.0.

## Additional Information

**How to cite this article**: Johnson-Beebout, S. E. *et al*. Zn uptake behavior of rice genotypes and its implication on grain Zn biofortification. *Sci. Rep.* **6**, 38301; doi: 10.1038/srep38301 (2016).

**Publisher’s note:** Springer Nature remains neutral with regard to jurisdictional claims in published maps and institutional affiliations.

## Supplementary Material

Supplementary Information

## Figures and Tables

**Table 1:** Correlation coefficients (*r*) between Zn concentration of different plant parts and DTPA-extractable soil Zn at various depths as influenced by water management and Zn fertilization (*n* = 16): Exp. 1.

Plant tissue Zn concentration												
	Brown rice		Polished Grain		Panicle		Flagleaf		Stem		Leaf	
*Soil depths (cm)*	0–2	2–10	0–2	2–10	0–2	2–10	0–2	2–10	0–2	2–10	0–2	2–10
*CF* (*DAT*)												
1–30									NS	NS	0.75**	NS
32–37									*0.45*	0.85**	0.55*	0.78**
86 –91	0.58*	NS	NS	NS	0.55*	*0.46*	0.60**	NS	0.72**	0.66**	*0.48*	*0.40*
113	NS	*0.48*	NS	*0.47*	NS	NS	0.57*	NS	NS	0.62**	NS	*0.43*
*MSD* (*DAT*)												
1–30									0.71**	NS	0.88**	ns
32 –37									0.70**	0.77**	0.57*	0.96**
86–91	NS	0.79**	*0.45*	NS	NS	NS	NS	NS	0.60**	NS	NS	NS
113	NS	NS	NS	NS	NS	NS	NS	NS	0.56*	NS	NS	NS
*LSD* (*DAT*)												
1–30									0.71**	NS	NS	NS
32 –37									0.69**	0.86**	0.81**	0.82**
86–91	0.70**	0.54*	0.59*	*0.48*	NS	NS	NS	NS	0.78**	0.61*	NS	NS
113	*0.44*	0.56*	*0.48*	*0.43*	NS	NS	NS	0.51*	NS	0.83**	NS	NS

*NS*, non–significant at 5% level.

*Significant at 5% level.

**Significant at 1% level.

*Italic* values without * are significant at 10% level;

*DAT*, days after transplanting; *CF*, continuous flooding; *MSD*, mid-season drying; *LSD*, late-season drying.

**Table 2:** Effects of genotypes, water management and Zn fertilization on Zn uptake of various rice plant parts at early heading: Exp. 2.

Treatments	Panicle (μg g^−1^)	Stem (μg g^−1^)	Flagleaf (μg g^−1^)	Leaf (μg g^−1^)	Deadleaf (μg g^−1^)
**Genotypes**					
*IR69428*	155 ± 8.00b	650 ± 59.0b	26.0 ± 1.40a	112 ± 5.00a	61.0 ± 11.0a
*IR68144*	196 ± 10.0a	820 ± 100a	27.0 ± 3.20a	50.0 ± 2.00b	27.0 ± 2.40b
**Water management**					
*Drying period 0*	178 ± 13.0a	614 ± 142b	22.0 ± 2.00a	75.0 ± 11.0b	36.7 ± 10.0a
*Drying period 1*	180 ± 15.0a	644 ± 111b	31.0 ± 7.00a	73.0 ± 11.0b	45.0 ± 9.00a
*Drying period 2*	191 ± 20.0a	855 ± 150a	26.0 ± 3.00a	84.0 ± 15.0b	53.0 ± 13.0a
*Drying period 3*	194 ± 10.0a	825 ± 137a	25.0 ± 2.00a	95.0 ± 14.0a	55.0 ± 25.0a
*Drying period 4*	133 ± 10.0b	737 ± 127ab	26.0 ± 2.00a	80.0 ± 11.0b	31.0 ± 7.00a
**Zinc Fertilization**					
*No Zn*	147 ± 12.0c	363 + 24.0d	23.0 ± 2.50b	68.5 ± 9.80b	18.0 ± 2.00b
*Zn at active tillering*	175 ± 10.0b	743 ± 49.0b	24.0 ± 2.00b	89.0 ± 14.0a	32.0 ± 5.00b
*Zn at panicle initiation*	208 ± 14.5a	1230 ± 91.0a	35.0 ± 5.00a	94.0 ± 10.0a	42.0 ± 4.00b
*Zn at early-heading*	171 ± 14.0b	604 ± 66.0c	23.7 ± 1.50b	74.0 ± 10.0b	83.0 ± 19.0a

Means in columns (per treatment) followed by the same letter are not significantly different from one another at *P* < 0.05. *Drying period 1*, 1 week drying before Zn application at active tillering; *Drying period 2*, 1 drying week following Zn application at active tillering; *Drying period 3*, 1 week drying before Zn application at panicle initiation; *Drying period 4*, 1 week drying after panicle initiation.

**Table 3:** Correlation coefficients (*r*) between Zn concentration/uptake in rice plant parts and DTPA-extractable soil Zn measured at different times for two genotypes, IR68144 and IR69428: Exp. 2.

	Zinc uptake (n = 20)						Zinc concentration (n = 20)						
	Grain	Panicle	Flagleaf	Stem	Leaf	Dead leaves	Grain	Panicle	Flagleaf	Stem	Leaf	Dead leaves	
**IR68144**													
Soil Zn 7–25 DAT				NS	*0.42*	NS				NS	NS	NS	
Soil Zn 26–47 DAT				NS	0.60**	NS				NS	NS	NS	
Soil Zn 69–75 DAT		NS	NS	NS	NS	NS		NS	NS	NS	NS	NS	
Soil Zn 83–110 DAT	NS	NS	NS	NS	NS	NS	NS	NS	NS	NS	NS	NS	
**IR69428**													
Soil Zn 7–25 DAT			NS	NS	0.46*	NS		NS	NS	NS	NS	NS	
Soil Zn 26–47 DAT			NS	0.52*	0.63**	NS		NS	NS	NS	0.50*	NS	
Soil Zn 69–75 DAT		0.53**	NS	0.66**	0.70**	NS		NS	NS	0.65**	0.53*	NS	
Soil Zn 83–110 DAT	0.53**		NS	0.67**	0.56*	0.49*	0.67**		0.60**	0.78**	0.46*	0.65**	

*NS*, Non significant at 5% level.

*Significant at 5% level.

**Significant at 1% level.

*Italic* values without * are significant at 10% level.

*DAT*, days after transplanting; *CF*, continuous flooding; *MSD*, mid-season drying; *LSD*, late-season drying.

**Table 4:** Correlation coefficients (*r*) between grain and other plant parts Zn concentration measured during early heading and maturity of two genotypes, IR68144 and IR69428: Exp. 2.

Plant parts	Grain Zn IR68144		Grain Zn IR69428	
	Uptake	Concentration	Uptake	Concentration
*Early heading* (*n* = 20)				
Flag leaf† Zn	NS	NS	NS	NS
Stem Zn	0.65**	0.81**	0.72**	0.67**
Leaf Zn	0.60**	0.61**	0.68**	NS
Dead leaves	NS	NS	0.47*	0.45*
*Maturity* (*n* = 20)				
Flag leaf Zn	0.72**	0.62**	0.60**	NS
Stem Zn	0.45*	0.62**	0.88**	0.82**
Leaf Zn	0.73**	0.64**	0.81**	0.59**
Dead leaves	0.63**	0.61**	0.72**	0.50*

*NS*, non-significant at 5% level.

*Significant at 5% level.

**Significant at 1% level.

^†^Flag leaf is a leaf attached to a panicle.
