# Supplementary material for: Zn uptake behavior of rice genotypes and its implication on grain Zn biofortification
Source: Sci Rep. 2016 Dec 2;6:38301. doi: 10.1038/srep38301 (PMC5133611; doi:10.1038/srep38301)
Supplement: Supplementary Information [file srep38301-s1.pdf]

Zn uptake behavior of rice genotypes and its implication on grain Zn biofortification

Sarah E. Johnson-Beebout[1], Johnvie B. Goloran[1], Francis H.C. Rubianes[1], Jack D.C. Jacob[2] & Oliver B. Castillo[3]

[1]Crop and Environmental Sciences Division, International Rice Research Institute DAPO Box 7777 Metro Manila, Philippines

[2]Department of Chemical and Biomolecular Engineering, University of Houston, Texas USA.

[3]APOTEX, Incorporated, Toronto, Ontario Canada

Supplementary Tables:

Table S1. ANOVA of DTPA-extractable soil Zn s at different depths Experiment 1.

			Soil depth	
			(0–2 cm)	(2-10 cm)
DAT	Treatment	df	*P* value	*P* value
1-30	WM	2	NS	NS
	ZF	3	0.0000***	0.0000***
	WM x ZF	6	NS	NS
30-37	WM	2	0.0000***	NS
	ZF	3	0.0000***	0.0000***
	WM x ZF	6	NS	NS
87-91	WM	2	0.0000***	0.0018**
	ZF	3	0.0000***	0.0000***
	WM x ZF	6	0.0000***	NS
113	WM	2	0.0129*	NS
	ZF	3	0.0000***	0.0011**
	WM x ZF	6	0.0250*	NS

DAT, day after transplanting; WM, water management regime; ZFM, Zinc fertilization management; *NS*, Non significant at 5% level.
* Significant at 5% level.
** Significant at 1% level.
*** Significant at 0.1% level of significance.

Table S2. ANOVA for Zn concentration and uptake (concentration x biomass) in different plant parts of rice measured at different stages of growth after transplanting: Experiment 1. Data are shown in Figure 3 and Tables S3 and S4.

Factors	df	Leaf	Flagleaf	Stem	Panicle	Brown rice	Polished Grain
				**Zinc uptake**			
**Growth period** (DAT)		P value	P value	P value	P value	P value	P value
1-30							
WM	2	NS		NS			
ZF	3	0.0000***		0.0000***			
WM*ZF	6	NS		NS			
32-37							
WM	2	NS		0.0089**			
ZF	3	0.0000***		0.0000***			
WM*ZF	6	NS		NS			
86-91							
WM	2	NS	NS	NS	0.0006***		
ZF	3	0.0384*	NS	0.0000***	0.0431*		
WM*ZF	6	0.0164*	NS	NS	0.0246*		
113							
WM	2	NS	0.0442	NS		0.0452*	0.0031**
ZF	3	0.0058**	NS	0.0000***		0.0384*	NS
WM*ZF	6	NS	NS	0.0037***		NS	NS
**Growth period**				**Zinc concentration**			
1-30							
WM	2	NS		NS			
ZF	3	0.0029**		0.0000***			
WM*ZF	6	NS		NS			
32-37							
WM	2	NS		NS			
ZF	3	0.0000***		0.0000***			
WM*ZF	6	NS		NS			
86-91							
WM	2	0.0086**	0.0352*	0.0082	NS		
ZF	3	0.0009***	NS	0.0000***	0.0028**		
WM*ZF	6	NS	0.0096**	NS	NS		
113							
WM	2	NS	NS	0.0111*		NS	0.0005***
ZF	3	0.0000***	NS	0.0000***		0.0000***	0.0076**
WM*ZF	6	NS	NS	0.0167*		NS	NS

DAT, day after transplanting; *WM*, water management; *ZF*, zinc fertilization.
Blank columns or rows indicate no data or not appropriate.
NS, Non significant at 5% level.
* Significant at 5% level.
** Significant at 1% level.
*** Significant at 0.1% level of significance.

Table S3. Mean and standard error of Zn concentration in various parts of rice plants as influenced by water management (WM) and Zn fertilization (ZF): Experiment 1.

Treatments	Panicle ($\mu g\ g^{-1}$)	Stem ($\mu g\ g^{-1}$)	Flagleaf ($\mu g\ g^{-1}$)	Leaf ($\mu g\ g^{-1}$)
**1-30 DAT**				
WM (*n* = 16)				
Continuous flooding		41.3 ± 1.26aA		30.8 ± 1.57aB
Mid-season drying		42.2 ± 0.82aA		31.5 ± 1.35aB
Late-season drying		42.1 ± 1.43aA		30.8 ± 1.00aB
ZF (*n* =12)				
No Zn		40.0 ± 1.20bA		30.6 ± 1.10bB
Bazal Zn		47.5 ± 0.95aA		35.7 ± 2.07aB
Mid-season Zn		40.3 ± 1.08bA		28.7 ± 0.64bB
Late-season Zn		39.5 ± 0.62bA		29.0 ± 0.92bB
**32-37 DAT**				
WM (*n* = 16)				
Continuous flooding		95.0 ± 22.0bA		40.9 ± 5.10aB
Mid-season drying		117 ± 22.9aA		45.8 ± 6.31aB
Late-season drying		100 ± 22.7abA		41.4 ± 6.60aB
ZF (*n* =12)				
No Zn		50.0 ± 2.10bA		29.6 ± 1.17bB
Bazal Zn		70.2 ± 4.52bA		33.4 ± 1.40bB
Mid-season Zn		247 ± 15.3aA		77.3 ± 7.03aB
Late-season Zn		49.3 ± 2.20bA		30.4 ± 1.50bB
**86-91 DAT**				
WM (*n* = 16)				
Continuous flooding	38.0 ± 1.62aB	46.2 ± 4.07bA		24.8 ± 0.49abC
Mid-season drying	40.2 ± 1.40aB	55.1 ± 4.49aA		26.5 ± 0.76aC
Late-season drying	37.0 ± 1.54aB	42.4 ± 1.77bA		23.7 ± 0.84bC
ZF (*n* =12)				
No Zn	36.5 ± 1.68bA	33.0 ± 2.62bA		23.8 ± 0.71bB
Bazal Zn	38.1 ± 1.21bB	51.4 ± 5.27aA		25.2 ± 0.77bC
Mid-season Zn	42.0 ± 1.61aB	57.6 ± 3.34aA		27.5 ± 0.86aC
Late-season Zn	37.0 ± 2.16bB	48.5 ± 2.47aA		23.5 ± 0.74bC
**113 DAT**				
WM (*n* = 16)				
Continuous flooding			19.6 ± 1.60aA	20.5 ± 1.31aA
Mid-season drying			21.3 ± 1.00aA	20.1 ± 0.87aA
Late-season drying			20.6 ± 1.54aA	21.2 ± 0.72aA
ZF (*n* =12)				
No Zn			20.2 ± 1.19aA	19.3 ± 0.60bcA
Bazal Zn			22.3 ± 1.79aA	21.7 ± 1.12abA
Mid-season Zn			17.4 ± 1.26aB	23.6 ± 1.38aA
Late-season Zn			22.3 ± 1.18A	17.9 ± 0.55cB

Means in columns (lowercase) or rows (uppercase) followed by the same letter are not significantly different from one another at $P < 0.05$. Data presented are only those significantly affected by individual treatment, and not those with significant interaction between treatments (WM x ZF).

Table S4. Mean and standard error of Zn uptake in various parts of rice plants as influenced by water management (WM) and Zn fertilization (ZF): Experiment 1

Treatments	Panicle (mg plant^{-1})	Stem (mg plant^{-1})	Flagleaf (mg plant^{-1})	Leaf (mg plant^{-1})
**1-30 DAT**				
WM (n = 16)				
Continuous flooding		96.1 ± 6.20aA		72.5 ± 5.80aB
Mid-season drying		102 ± 7.70aA		76.4 ± 5.50aB
Late-season drying		94.1 ± 7.60aA		69.2 ± 4.90aB
ZF (n =12)				
No Zn		88.2 ± 5.20bA		67.0 ± 2.90bB
Bazal Zn		131 ± 9.10aA		100 ± 6.60aB
Mid-season Zn		88.1 ± 3.60bA		63.1 ± 2.90bB
Late-season Zn		81.8 ± 4.40bA		60.6 ± 3.10bB
**32-37 DAT**				
WM (n = 16)				
Continuous flooding		335 ± 74.5cA		165 ± 23.0aB
Mid-season drying		393 ± 76.3aA		169 ± 18.9aB
Late-season drying		364 ± 88.9bA		171 ± 35.0aB
ZF (n =12)				
No Zn		163 ± 11.4cA		109 ± 6.50bB
Bazal Zn		257 ± 20.8bA		142 ± 8.30bB
Mid-season Zn		865 ± 59.7aA		310 ± 34.3aB
Late-season Zn		170 ± 12.9cA		112 ± 8.90bB
**86-91 DAT**				
WM (n = 16)				
Continuous flooding		1126 ± 103aA	72.2 ± 3.80bB	
Mid-season drying		1292 ± 142aA	74.9 ± 7.50aB	
Late-season drying		1100 ± 71.9aA	72.9 ± 5.70bB	
ZF (n =12)				
No Zn		764 ± 63.0bA	65.3 ± 8.60aB	
Bazal Zn		1304 ± 170aA	81.5 ± 5.20aB	
Mid-season Zn		1413 ± 96.0aA	68.9 ± 6.10aB	
Late-season Zn		1211 ± 64.4aA	77.6 ± 5.80aB	
**113 DAT**				
WM (n = 16)				
Continuous flooding			56.8 ± 5.40aB	201 ± 13.6aA
Mid-season drying			60.5 ± 3.30aB	209 ± 11.6aA
Late-season drying			72.3 ± 6.10aB	203 ± 7.80aA
ZF (n =12)				
No Zn			62.0 ± 4.90B	189 ± 6.20bA
Bazal Zn			72.2 ± 6.20B	201 ± 12.5bA
Mid-season Zn			58.0 ± 7.40B	243 ± 12.7aA
Late-season Zn			60.4 ± 5.30B	184 ± 12.1bA

Means in columns (lowercase) or rows (uppercase) followed by the same letter are not significantly different from one another at $P < 0.05$. Data presented are only those significantly affected by individual treatment, and not those with significant interaction between treatments (WM x ZF).

Table S5. ANOVA of Fe and P concentration in different parts of rice measured from early stage of growth to maturity: Experiment 1. Data are shown in Figure 4.

DAT	Treatment	Grain (*P* value)		Panicle (*P* value)		Stem (*P* value)		Leaf (*P* value)		Flagleaf (*P* value)	
		Fe	P	Fe	P	Fe	P	Fe	P	Fe	P
30	WM					NS	NS	NS	NS		
	ZF					NS	NS	NS	NS		
	WM x ZF					NS	NS	NS	0.0394*		
37	WM					NS	NS	NS	NS		
	ZF					NS	0.0000***	NS	NS		
	WM x ZF					NS	0.0441*	NS	NS		
91	WM			NS	NS	NS	0.0011**	NS	0.0000***	NS	NS
	ZF			NS	0.0224*	NS	NS	NS	NS	0.0036**	NS
	WM x ZF			NS	NS	NS	0.0073	NS	NS	NS	NS
113	WM	NS	0.0061**	NS	NS	NS	0.0009***	NS	0.0000***	NS	NS
	ZF	NS	0.0000***	NS	NS	NS	NS	NS	NS	NS	NS
	WM x ZF	NS	0.0311*	NS	NS	NS	0.0475*	NS	NS	NS	NS

WM, water management; *ZF*, zinc fertilization; *P*, phosphorus.Blank columns or rows indicate no data or not applicable for that plant parts.
NS, Non significant at 5% level.
* Significant at 5% level.
** Significant at 1% level.
*** Significant at 0.1% level of significance.

Table S6. ANOVA of DTPA-extractable soil Zn measured at different times[1] after planting for experiment 2. Data are shown in Figures 5 and S1.

Time	Treatment	*P* value
Early tillering	WM	NS
(7-25 DAT)[1]	ZF	NS
	GT	NS
	WM*ZF	NS
	WM*GT	NS
	ZF*GT	0.0045**
	WM*ZF*GT	NS
Active tillering	WM	NS
(26-47 DAT)	ZF	0.0047**
	GT	NS
	WM*ZF	NS
	WM*GT	NS
	ZF*GT	0.0079**
	WM*ZF*GT	NS
Flowering	WM	NS
(69-75 DAT)	ZF	0.0097**
	GT	NS
	WM*ZF	NS
	WM*GT	NS
	ZF*GT	0.0481*
	WM*ZF*GT	NS
Grain filling	WM	NS
(83-110 DAT)	ZF	0.0176*
	GT	NS
	WM*ZF	NS
	WM*GT	NS
	ZF*GT	NS
	WM*ZF*GT	NS

[1] Means of each group for DTPA-extractable soil Zn to represent the available soil Zn for that particular period or stage of rice growth (See statistical analyses). *WM* water management, *GT* genotype, *ZF* zinc fertilization management, *DAT*, days after planting.
NS, Non significant at 5% level.
* Significant at 5% level.
** Significant at 1% level.
*** Significant at 0.1% level of significance.

Table S7. ANOVA for Zinc uptake and concentration in different plant parts of at different stages of growth.

Factors	df	Brown rice Zn	Panicle	Leaf	Flagleaf	Dead leaves	Stem
**Zinc uptake**		P value	P value	P value	P value	P value	P value
Early-heading							
WM	4		0.0047**	0.0110*	NS	NS	0.0110*
ZF	3		0.0025**	0.0000***	0.0330*	0.0001***	0.0000***
GT	1		0.0005***	0.0250*	NS	0.0002***	0.0250*
WM*ZF	12		NS	NS	NS	NS	NS
WM*GT	4		NS	NS	NS	NS	NS
ZF*GT	3		NS	NS	NS	NS	NS
WM*ZF*GT	12		NS	NS	NS	NS	NS
Maturity							
WM	4	NS		0.0146*	ns	ns	0.0128*
ZF	3	0.0005***		0.0035**	0.0121*	0.0005***	0.0000***
GT	1	0.0015***		0.0000***	0.0001***	0.0001***	0.0033**
WM*ZF	12	NS		NS	NS	NS	0.0431*
WM*GT	4	NS		NS	NS	NS	NS
ZF*GT	3	NS		NS	NS	NS	0.0050**
WM*ZF*GT	12	NS		NS	NS	NS	NS
**Zinc concentration**							
Early-heading							
WM	4		NS	NS	NS	NS	NS
ZF	3		0.0001***	0.0000***	0.0081**	0.0000***	0.0000***
GT	1		0.0000***	0.0000***	0.0000***	0.0023**	0.0000***
WM*ZF	12		NS	NS	NS	NS	NS
WM*GT	4		NS	NS	NS	NS	NS
ZF*GT	3		NS	NS	NS	0.0002***	NS
WM*ZF*GT			NS	NS	NS	NS	NS
Maturity							
WM	12	0.0133*		NS	NS	NS	NS
ZF		0.0000***		0.0000***	0.0001***	0.0001***	0.0000***
GT	1	0.0000***		0.0125*	NS	NS	0.0000***
WM*ZF	12	NS		NS	NS	NS	NS
WM*GT	4	0.0013**		NS	NS	NS	NS
ZF*GT	3	0.0166*		NS	NS	0.0059***	NS
WM*ZF*GT	12	NS		NS	NS	NS	NS

DAT, days after transplanting; *WM*, water management; *ZF*, zinc fertilization; *NS*, Non significant at 5% level.
* Significant at 5% level;** Significant at 1% level;*** Significant at 0.1% level of significance.
